# Spatiotemporal Accuracy of Gradient Magnetic-Field Topography (GMFT) Confirmed by Simultaneous Magnetoencephalography and Intracranial Electroencephalography Recordings in Patients with Intractable Epilepsy

**DOI:** 10.3389/fncir.2016.00065

**Published:** 2016-08-19

**Authors:** Hiroshi Shirozu, Akira Hashizume, Hiroshi Masuda, Masafumi Fukuda, Yosuke Ito, Yoko Nakayama, Takefumi Higashijima, Shigeki Kameyama

**Affiliations:** ^1^Department of Functional Neurosurgery, Nishi-Niigata Chuo National HospitalNiigata, Japan; ^2^Department of Neurosurgery, Takanobashi Central HospitalHiroshima, Japan

**Keywords:** magnetoencephalography, gradient magnetic-field topography, spatiotemporal resolution, simultaneous recording, intracranial electroencephalography, voltage topography

## Abstract

Gradient magnetic-field topography (GMFT) is one method for analyzing magnetoencephalography (MEG) and representing the spatiotemporal dynamics of activity on the brain surface. In contrast to spatial filters, GMFT does not include a process reconstructing sources by mixing sensor signals with adequate weighting. Consequently, noisy sensors have localized and limited effects on the results, and GMFT can handle MEG recordings with low signal-to-noise ratio. This property is derived from the principle of the planar-type gradiometer, which obtains maximum gradient magnetic-field signals just above the electrical current source. We assumed that this characteristic allows GMFT to represent even faint changes in brain activities that cannot be achieved with conventional equivalent current dipole analysis or spatial filters. GMFT is thus hypothesized to represent brain surface activities from onset to propagation of epileptic discharges. This study aimed to validate the spatiotemporal accuracy of GMFT by analyzing epileptic activities using simultaneous MEG and intracranial electroencephalography (iEEG) recordings. Participants in this study comprised 12 patients with intractable epilepsy. Epileptic spikes simultaneously detected on both MEG and iEEG were analyzed by GMFT and voltage topography (VT), respectively. Discrepancies in spatial distribution between GMFT and VT were evaluated for each epileptic spike. On the lateral cortices, areas of GMFT activity onset were almost concordant with VT activities arising at the gyral unit level (concordance rate, 66.7–100%). Median time lag between GMFT and VT at onset in each patient was 11.0–42.0 ms. On the temporal base, VT represented basal activities, whereas GMFT failed but instead represented propagated activities of the lateral temporal cortices. Activities limited to within the basal temporal or deep brain region were not reflected on GMFT. In conclusion, GMFT appears to accurately represent brain activities of the lateral cortices at the gyral unit level. The slight time lag between GMFT and VT is likely attributable to differences in the detection principles underlying MEG and iEEG. GMFT has great potential for investigating the spatiotemporal dynamics of lateral brain surface activities.

## Introduction

Magnetoencephalography (MEG) is a noninvasive examination used for neurophysiological evaluations. Because of the advantageous spatial resolution (Gharib et al., 1995), MEG is often used for localization of brain functions and epileptic foci (Knowlton et al., 1997; Stefan et al., 2003). Source localization of cortical activities is conventionally estimated by equivalent current dipole (ECD), which can simply represent the sources of brain activities, and is often used for evoked activities such as somatosensory, auditory, and visual evoked magnetic fields (Baumgartner et al., 1991; Nakasato et al., 1995; Seki et al., 1996; Nakasato and Yoshimoto, 2000; Onishi et al., 2013). ECD is also useful in evaluating epilepsy. Localization of focal epileptic activities by ECD is clinically valuable, particularly in patients with medically intractable epilepsy who are considered as candidates for surgical treatment (Otsubo et al., 2001a; Pataraia et al., 2002; Oishi et al., 2006).

Although ECD estimation is a valuable method, some problems remain. Despite the fact that meaningful cortical activity commonly involves synchronous activation of a circumscribed area, ECD can only show a center of gravity of the cortical activity at one time point (Pataraia et al., 2002). One study on epileptic activities revealed that an area of cortex wider than 3 cm^2^ was necessary to produce detectable magnetic spikes on MEG (Oishi et al., 2002). Moreover, approximating activities with relatively complicated spatiotemporal dynamics is physiologically and mathematically difficult using only one ECD. For example, representing the totality of dynamic changes in distributions with activities such as language function (Gow and Caplan, 2012) and epileptic activities (Alarcon et al., 1997; Spencer, 2002) is difficult using only one ECD at one time point. To solve these problems, various spatial filters have been developed. Spatial filters such as minimum norm estimation (Molins et al., 2008; Tanaka et al., 2010) and others (Kirsch et al., 2006; Ishii et al., 2008) can allow delineation of the spatial distribution of brain activities. However, both ECD and spatial filters have a crucial problem in MEG analyses, in that a solution to the biomagnetic inverse problem is required. In general, both techniques require adequate signal-to-noise ratio (SNR) in the intended data for physiologically reasonable source reconstruction (Bowyer et al., 2003).

Gradient magnetic-field topography (GMFT) is an alternative method that can represent the spatiotemporal dynamics of cortical activities in a similar manner to other spatial filters. GMFT produces a color-coded representation of the magnetic-field gradient on the individual 3-dimensional (3D) rendered brain surface at each time point during brain activity. In past reports, GMFT has been applied to preoperative analyses of patients with intractable localization-related epilepsy (Hashizume et al., 2007; Shirozu et al., 2010). Unlike spatial filters, GMFT does not need to solve the biomagnetic inverse problem. This is derived from the process of generating GMFT, which is based on the characteristics of the planar gradiometer. Because the maximum gradient magnetic-field as measured by planar gradiometer is just above the electrical source (Ahonen et al., 1993; Hämäläinen et al., 1993), the distribution of the power of the gradient magnetic-field on each sensor is thought to correlate to the electrical voltage distribution of the cortical activity. Although ECD estimation often fails to interpret the rising phase of epileptic spikes with relatively high statistical reliability, GMFT never fails, because this method offers a completely faithful representation of any signals obtained from planar gradiometers.

The present study aimed to validate the spatiotemporal accuracy of GMFT. For this purpose, we performed simultaneous recordings of MEG and intracranial electroencephalography (iEEG) in patients with intracranial epilepsy, and compared the distribution of GMFT and actual electrical voltage of epileptic activity. We hypothesized that the distributions of GMFT and electrical voltage were concordant in those epileptic spikes recorded simultaneously on MEG and iEEG.

## Materials and methods

### Patients

This study enrolled 12 patients (6 females, 6 males) with medically intractable epilepsy who underwent resective surgery using extraoperative iEEG evaluation between August 2011 and April 2013. Median age at surgery was 14.0 years old (range, 3–36 years). Epilepsy diagnoses included frontal lobe epilepsy (*n* = 4), parietal lobe epilepsy (*n* = 3), lateral temporal lobe epilepsy (*n* = 3), and medial temporal lobe epilepsy (*n* = 2). All patients underwent simultaneous recordings of MEG and iEEG in a magnetically shielded room during chronic extraoperative iEEG monitoring. We implanted subdural, 0.8-mm-thick silicone-embedded, platinum grid, and strip electrodes (10 mm intercontact distance; Unique Medical, Tokyo, Japan), and added depth electrodes if necessary to investigate deeper areas of the brain. Determinations of implantation area were based on the results of preoperative evaluation including seizure semiology, EEG, magnetic resonance imaging (MRI), single-photon emission computed tomography (SPECT), and MEG. Preoperative MEG analyses were analyzed using conventional ECD analyses. Resection areas were decided on the results of chronic extraoperative iEEG. Patient profiles are described in Table 1.

**Table 1:** **Patient profiles**.

**Case**	**Age**	**Sex**	**Epilepsy**	**Pathology**	**Implanted IEs**						**IEs for simultaneous recording of MEG and iEEG**					
					**Total no**.	**Lateral surface**	**DE**	**BE**	**IH**	**mT**	**Recorded electrode no**.	**Recorded DE**	**Recorded BE**	**Recorded IH**	**Recorded mT**	**Electrodes for VT**
1	17	M	Lt.PLE	FCD type I	112	88	24	0	0	0	32	12	–	–	–	20
2	29	M	Rt.FLE	FCD type I	192	108	24	T16, F16	20	8	60	0	4	0	0	58
3	3	F	Lt.TLE	TS	122	72	18	T32	0	0	60	0	32	–	–	60
4	13	F	Rt.TLE	Astrocytoma	44	16	0	T20	0	8	44	–	20	–	8	16
5	11	M	Lt.PLE	FCD type IIb	114	90	24	0	0	0	60	15	-	–	–	45
6	5	M	Lt.FLE	FCD type I	112	80	24	F8	0	0	60	16	8	–	–	42
7	15	F	Rt.TLE	TS	114	66	24	T16	0	8	60	24	8	–	0	32
8	19	F	Lt.TLE	DNT	82	38	18	T18	0	8	60	6	9	–	4	48
9	12	F	Lt.TLE	NS (Abn.Hp.Gy)	60	16	0	LT16, RT20	0	LT8	60	–	LT16, RT12	–	LT8	LT28, RT18
10	31	M	Rt.FLE	FCD type IIb	110	72	24	F8	6	0	60	20	4	3	–	38
11	36	M	Rt.PLE	DT	156	116	0	O8, T8	24	0	60	–	O4, T4	12	–	40
12	13	M	Rt.FLE	FCD type I	142	104	24	F8	6	0	60	12	4	4	–	44

*Abn.Hp.Gy, abnormal hippocampal gyration; BE, electrodes on frontal or temporal base regions; DE, depth electrodes; DNT, dysembrioplastic neuroepithelial tumor; DT, destructive tissue; FCD, focal cortical dysplasia; FLE, frontal lobe epilepsy; IE, intracranial electrodes; iEEG, intracranial electroencephalography; IH, electrodes on interhemispheric regions; Lt., left; LT, left temporal; M, male; MEG, magnetoencephalography; mT, electrodes on medial temporal regions; NS, no significant alterations; PLE, parietal lobe epilepsy; Rt., right; RT, right temporal; TLE, temporal lobe epilepsy; TS, tuberous sclerosis*.

All patients or their parents provided written, informed consent before each surgical treatment. The review board of our institute provided approval for the protocol of this retrospective study (No. 1532).

### Simultaneous MEG-iEEG recordings

Simultaneous MEG-iEEG recording was performed on the last day of chronic extraoperative iEEG monitoring. MEG was performed using a 306-channel (204-channel planar gradiometer, 102-channel magnetometer), whole-head-type neuromagnetometer (Neuromag System; Elekta-Neuromag Oy, Helsinki, Finland) at sampling rate of 600.615 Hz and with a band-pass filter of 0.1–200 Hz. The same Neuromag System was used to record iEEG. Due to the limitations of the Neuromag System, up to 60 channels of interest were selected and connected to the EEG port of the same neuromagnetometer system. The reference electrode was chosen from one implanted electrode other than the electrodes of interest mentioned above. The sampling rate and band-pass filter of iEEG were the same as those of MEG. To avoid motion artifacts from metallic materials, connectors of electrode-lead wires of iEEG were fixed to the sensor-helmet with adhesive tape, to avoid changes with body movement. To prevent unnecessary magnetization, we also took care not to perform postoperative MRI until completion of this simultaneous recording. Recording times comprised 4–6 sessions of 3–5 min each. Patients were given mild sedation with intravenous injection of thiopental or oral administration of pentobarbital to induce light sleep stage for enhancement of epileptic spikes.

### Spike detection and classification

Drowsy periods with relatively low noise and lasting 200–600 s were identified for analysis. Raw data from MEG were preprocessed by principal component analysis (PCA) to reduce noise, if necessary. On PCA preprocessing, minimal manipulation was carefully performed to avoid excessive reductions in actual cortical activity. Interictal epileptic spikes were visually collected with cross-referencing to MEG and iEEG. Detected spikes were counted and classified into three groups: spike ME, as spikes detected on both MEG and iEEG; and spike M and spike E, as spikes detected only on MEG or iEEG, respectively. Spike E were sub-analyzed by spike localization, i.e., localized to the lateral surface, deep brain area, basal or medial temporal area, interhemispheric area, or broad area. Spike ME were selected for further analysis of localization as described below.

### Analysis of MEG by GMFT

The data from the 204 channels of the planar gradiometer were used for GMFT analysis. GMFT was calculated using MATLAB-based free software (hns_meg; http://meg.aalip.jp). Signals from planar gradiometers detecting longitudinal or latitudinal gradients of magnetic fluxes are squared and summed at 102 sensor points. The 102 sensor signals calculated are projected onto individual cortical surfaces just beneath the sensors. Projected signal values were smoothed using a nearest-neighbor interpolation. The rationale of GMFT has been described elsewhere (Hashizume et al., 2007). In this study, MEG data were filtered at 5–45 Hz for GMFT. A 200-ms period including the rising phase of spike ME was analyzed and displayed serially in 2-ms intervals. Areas exceeding 200 fT/cm were determined as activated areas to distinguish from background activity, and the first activated area was considered as the onset of the spike.

### Analysis of iEEG by voltage topography

Analysis of iEEG by voltage topography (VT) was performed using a custom-made MATLAB-based program, similar to a previously described method (Otsubo et al., 2001b). In this study, VT represented the activated area by the absolute value of electric voltage with a band-pass filter of 5–45 Hz, identical to the filters used in MEG analysis. Electrodes placed on the cortical surface were superimposed onto the individual 3D-rendered MRI (iPlan Cranial 3.0: BRAINLAB, Feldkirchen, Germany) to create serial 2-ms intervals, the same as on GMFT. Channels for depth electrodes were not included for VT.

### Comparison of GMFT and VT

GMFT and VT were compared in terms of both spatial distribution and time difference. Spatial distribution was visually compared in the relationship of cerebral sulci and gyral-structures. Time lags were judged at the onset of activated areas on both GMFT and VT.

### Statistics

Statistical analyses were performed using SPSS (PASW Statistics 18; IBM, Chicago, IL, USA). Group mean comparisons of the number of detected spikes in spike ME, M and E were analyzed by Kruskal–Wallis test. Values of < 0.05 were considered statistically significant.

## Results

### Profiles of intracranial electrodes implantation

The median number of channels of implanted intracranial electrodes (IE) was 113.0 (range, 44–192). Depth electrodes were added in nine patients. IEs were placed on the frontal or temporal basal cortices in 10 patients, on the interhemispheric brain surfaces in 4, and on the medial temporal cortex in 5. The details of electrodes are described in Table 1.

### Spike detection

Detected spikes are described in Table 2. The median number of detected spike ME, spike M and spike E was 6 (range, 1–11), 2.5 (range, 0–13), and 41.5 (range, 3–235), respectively. Spike E was detected significantly more often than spike ME (*p* < 0.01) and spike M (*p* < 0.01). According to sub-analyses of spike E, all but Case 5 showed more localized spikes than broad spikes. In Case 5, 234 of the 235 spikes were noted in the broad area. The median number of spike E localized on the lateral surface was 5 (range, 0–189). In seven patients with depth electrodes and 10 patients with IEs over the basal or medial temporal brain surfaces, IEs showed 0–142 spikes (median, 0) and 0–159 spikes (median, 0.5), respectively. In four patients with IEs covering the interhemispheric surfaces, only one patient (Case 12) had six spikes.

**Table 2:** **Spike detection**.

**Case**	**Preprocessing**	**Analyzed time (s)**	**Spike ME**	**Spike M**	**Spike E**					
					**Total**	**Localized in lateral surface**	**Localized in deep brain area**	**Localized in basal or medial temporal area**	**Localized in interhemispheric area**	**Broad area**
1	PCA	300	11	0	168	12	142	NA	NA	14
2	PCA	200	4	2	10	10	NA	0	0	0
3	PCA	300	6	3	166	4	NA	159	NA	3
4	PCA	300	1	0	19	0	NA	16	NA	3
5	PCA	300	11	0	235	0	1	NA	NA	234
6	PCA	600	6	8	11	2	4	1	NA	4
7	PCA	200	5	1	59	37	0	0	NA	22
8	PCA	300	7	13	24	3	0	11	NA	10
9	None	300	1	Lt.1, Rt.1	65	Rt.2	NA	Lt.57, Rt.5	NA	Lt.1
10	None	200	7	0	189	189	0	0	0	0
11	None	240	4	8	3	3	NA	0	0	0
12	PCA	300	7	9	12	6	0	0	6	0

*Lt., left; NA, not available; PCA, principal component analysis; Rt., right; spikes detected on electrodes placed on frontal or temporal regions; spikes detected on depth electrodes; Spike E, spikes detected on intracranial electroencephalography (iEEG); spikes detected on electrodes placed on interhemispheric region; spikes detected only on electrodes placed on lateral cortices; Spike M, spikes detected only on MEG; Spike ME, spikes detected on both MEG and iEEG; spikes detected on electrodes placed on medial temporal region*.

### Comparison of GMFT and VT

**Figure 1:**
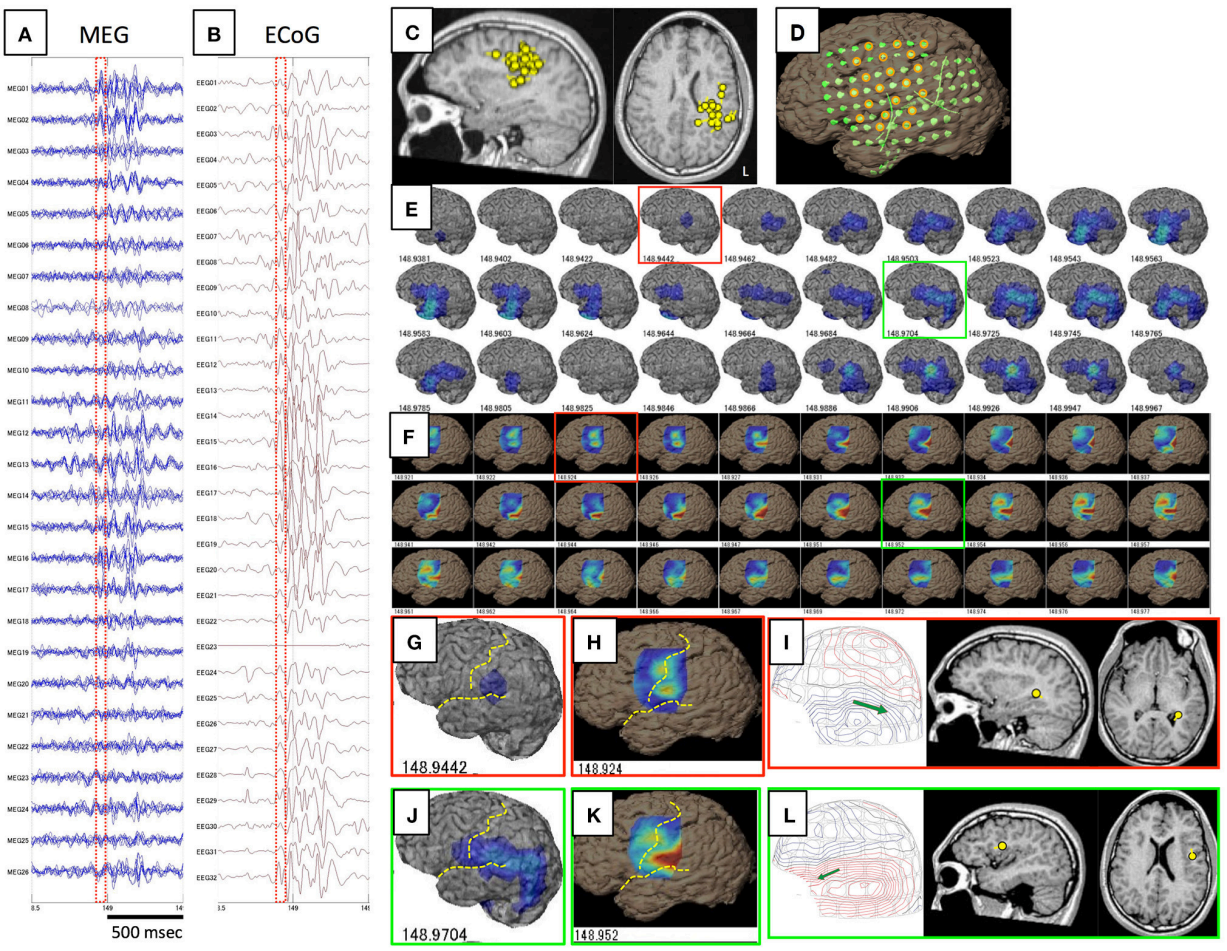
**This figure shows a comparison of GMFT and VT in Case 1**. Spike ME was detected simultaneously on both MEG **(A)** and iEEG **(B)**. This case had ECD cluster in the left parietal lobe **(C)** and intracranial electrodes were implanted over the frontal, parietal, and temporal lobes **(D)**. Some electrodes were selected for voltage topography (orange circles). A 60-ms part (red dot squares in **A,B**) of GMFT **(E)** and VT **(F)** demonstrated temporal changes in brain surface activities. Time intervals of GMFT and VT are 2 ms, and figures below each 3D-brain images indicate the temporal course. Activity starting on the supramarginal gyrus (red squares) is recognized on both GMFT **(G)** and VT **(H)**. Note that activity on VT (148.924) precedes that on GMFT (148.944). Brain activities propagate posteriorly to the angular gyrus (green circles) as shown by both GMFT **(J)** and VT **(K)**. However, VT cannot show the entire dynamics due to the limited area of electrodes employed for VT. Yellow dotted lines imply the central sulcus and Sylvian fissure. ECDs corresponding to each time point (red and green circles) are demonstrated in **(I,L)**. The isocontour map in the early phase is ambiguous and ECD is estimated in the deep area of the temporal lobe **(I)**. Late-phase ECD is localized to the frontal operculum, corresponding to the anterior part of the activated area shown on GMFT and VT **(L)**.

**Figure 2:**
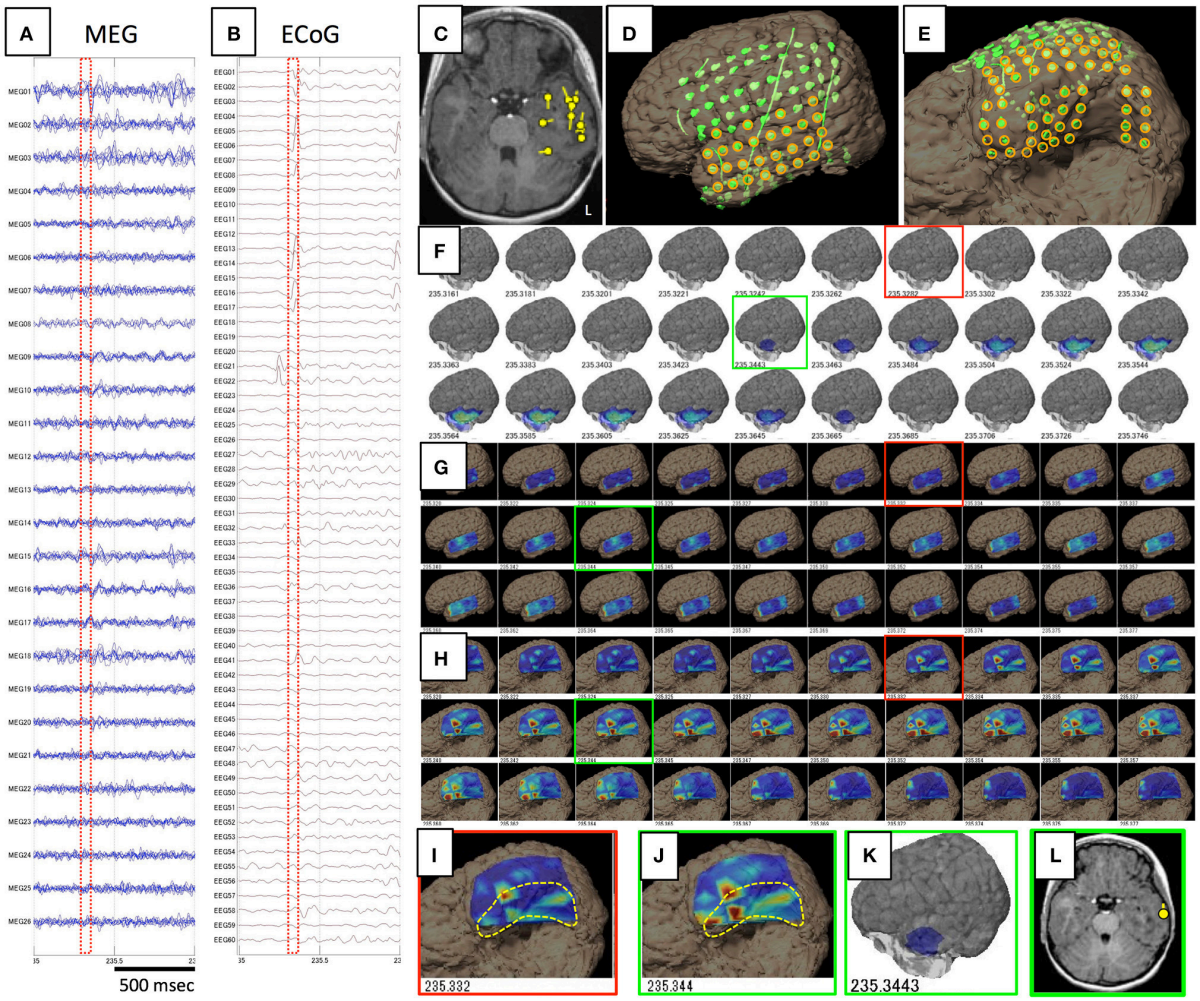
**Data and results for Case 3**. **(A,B)** show raw data for spike ME in MEG and iEEG, respectively. This case was estimated to show a broad epileptic focus in the left temporal lobe on conventional ECD analysis **(C)**. Intracranial electrodes were placed not only in the temporal, frontal, and parietal lobes **(D)**, but also on the temporal basal area **(E)**. Orange circles are used for VT on the lateral analysis **(G)** and basal analysis **(H)**. Although GMFT demonstrates brain surface activity in the anterior part of the left middle and inferior temporal gyri from 235.344 **(F,K)**, actual brain activity starts in the medial and basal temporal areas as displayed on VT of the temporal basal aspect **(H,I)**. For the first time when activity propagated to the inferior temporal gyrus **(J)**, GMFT could show the activated area **(G,K)**. Circumscribed areas with yellow dotted lines indicates the temporal base area, which cannot be seen from the lateral view. ECD in the late phase (green squares) can be estimated in the lateral temporal cortex **(L)**, whereas meaningful ECD is not estimated in the early phase (red squares).

**Figure 3:**
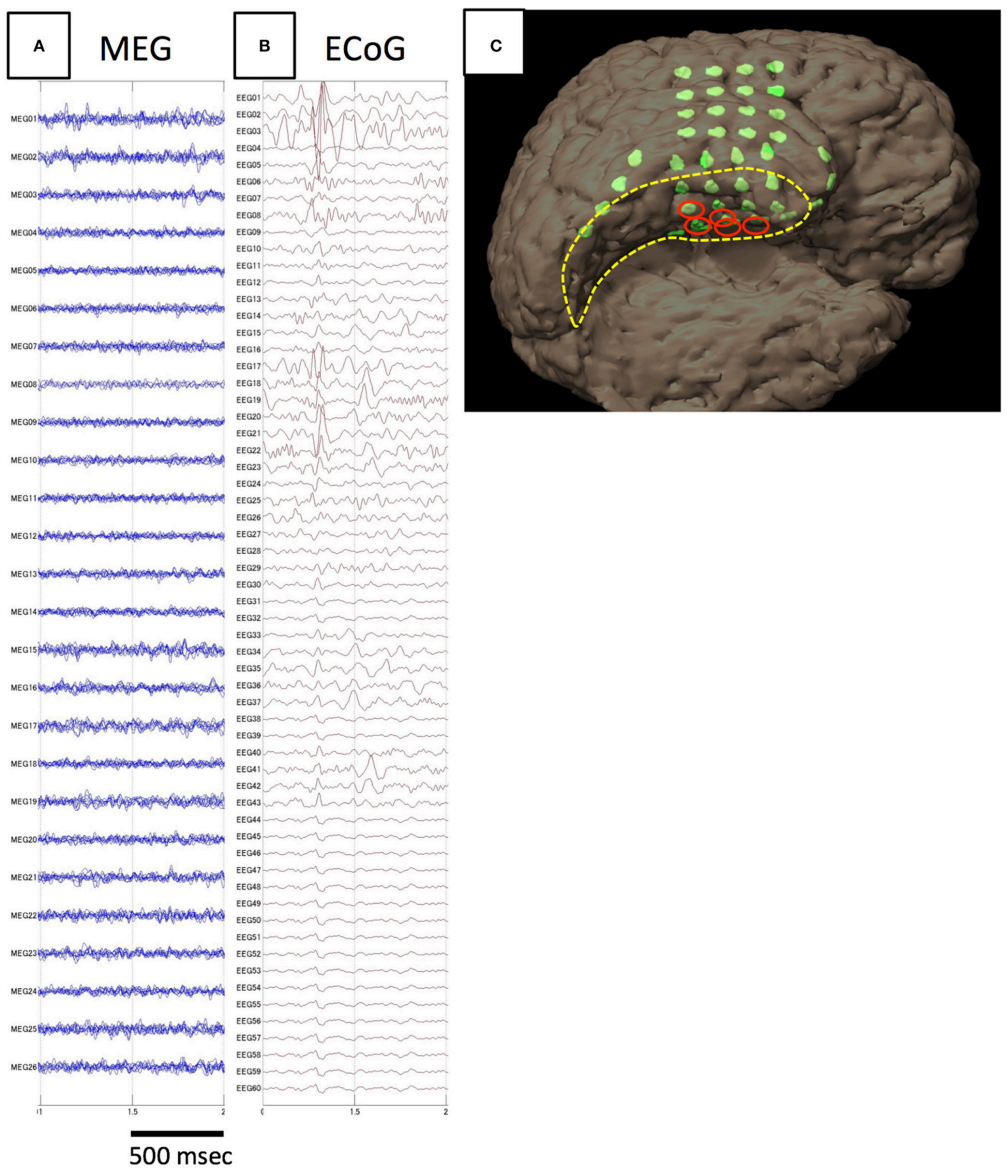
**Analysis of spike E identified in Case 4**. The spike is not detected on MEG **(A)**, but only on iEEG **(B)**. The electrodes from which spike E was recognized are demonstrated in the figure showing electrode locations on the individual 3D-rendered MRI as red ellipses **(C)**. Electrodes for spike E are localized to the medial temporal region.

**Figure 4:**
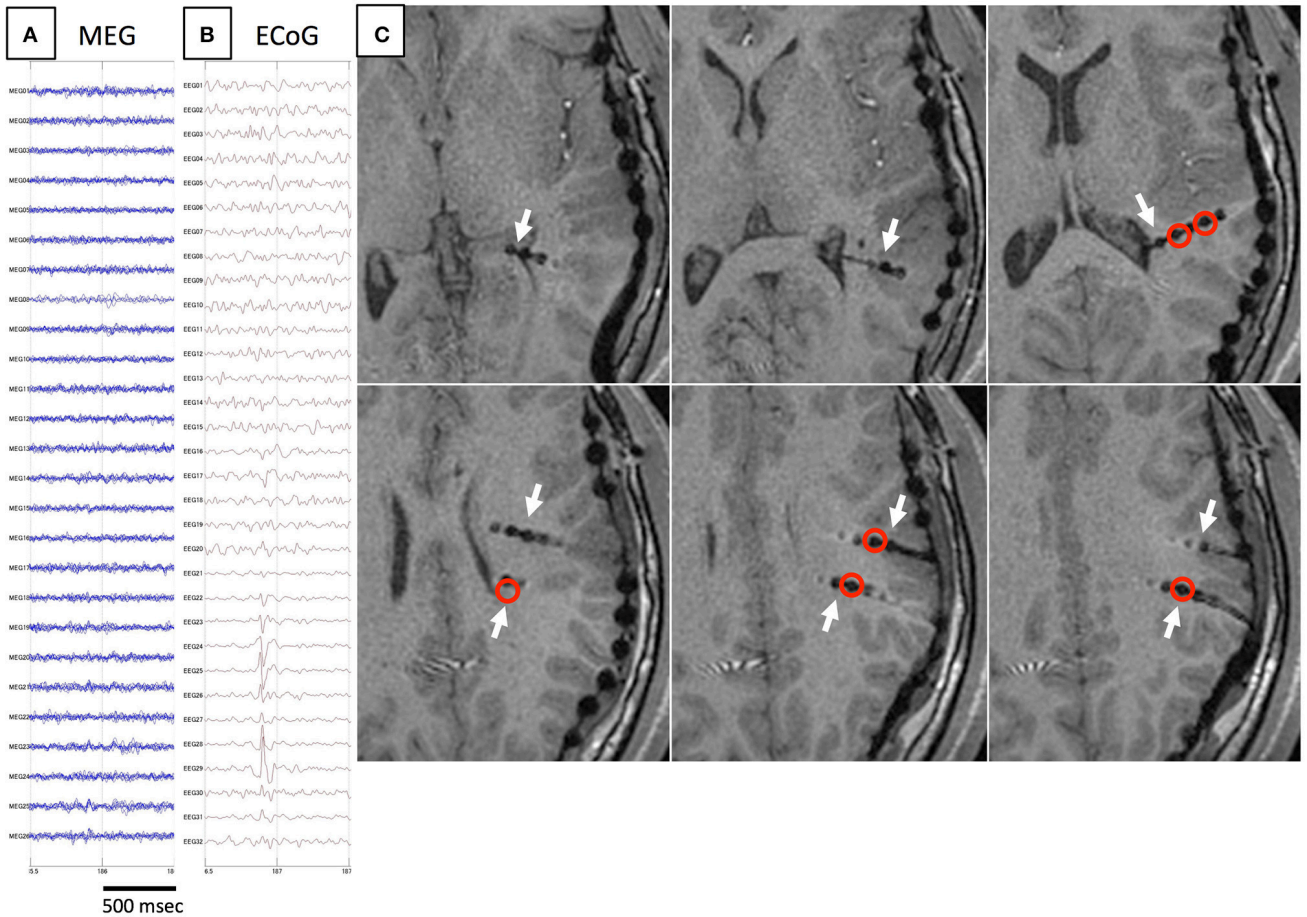
**Another example of spike E**. As in Figure 3, spikes are not detected on MEG **(A)**, but only on iEEG **(B)**. Axial MRI **(C)** demonstrates the location of depth electrodes (white arrows). Electrodes from which spike E are recognized are located in deep brain areas (red circles).

The results of comparisons are described in Table 3. In all patients, onset areas of GMFT were consistent with the epileptic foci decided from all examinations, semiology and surgical outcomes. Eight patients (66.7%) showed an excellent concordance rate (>80%) between GMFT and VT, and two patients (16.7%) showed good concordance (>60%). Spatial resolution was concordant at the gyral unit level (Figure 1). The remaining two patients did not present good concordance for the following reasons. Case 3 had a broad epileptic focus in the left temporal lobe, and some epileptic spikes emerged from the temporal basal region on iEEG. During simultaneous recording, four of the total six spike MEs originated in the temporal basal area and were displayed on VT but not GMFT at the onset of activity. GMFT showed spike activity when the activity propagated to the lateral surface (Figure 2). Similarly, onset of GMFT in the posterior part of the inferior temporal lobe did not match the onset of VT in the medial temporal region in only one spike ME for Case 4 (Figure 3). In patients with depth electrodes, three patients showed some localized spikes only seen on those depth electrodes. As a matter of course, these deeply limited activities were not demonstrated on GMFT (Figure 4).

**Table 3:** **Comparison of GMFT and VT**.

**Case**	**Onset area**		**Spatial concordance (%)**	**Preceding activation GMFT vs. VT (%)**	**Time lag at onset between GMFT and VT (median), ms**
	**GMFT**	**VT**			
1	iC, SMG, pSTG, IFG	iC, SMG, pSTG	9/11 (81.8)	VT, 11/11 (100)	3–38 (18.0)
2	MFG, IFG	MFG, IFG	4/4 (100)	VT, 4/4 (100)	14–37 (21.5)
3	MTG, ITG, pSTG, MFG	MTG, pSTG, Tbase	2/6 (33.3)	VT, 4/6 (66.6)	3–80 (12.5)
4	pITG	mT	0/1 (0)	VT, 1/1 (100)	36 (36.0)
5	ITG, pSTG, AG, SMG, iC	pSTG, AG, SMG, iC	10/11 (90.9)	VT, 11/11 (100)	0–40 (15.0)
6	IFG, iC, SMG, pSTG	IFG, iC, pSTG	4/6 (66.7)	VT, 6/6 (100)	1–81 (16.5)
7	MFG, iC, pSTG	MFG, iC, pSTG	4/5 (80.0)	VT, 5/5 (100)	8–41 (11.0)
8	pSTG	pSTG	1/1 (100)	VT, 1/1 (100)	21 (21.0)
9	ITG, MTG, STG	ITG, MTG, STG	7/7 (100)	VT, 7/7 (100)	8–69 (26.0)
10	SFG, MFG, iC	SFG, MFG, iC	6/7 (85.7)	VT, 5/7 (71.4)	8–31 (26.0)
11	sC	sC, iC, SPL	3/4 (75.0)	VT, 4/4 (100)	5–66 (42.0)
12	MFG, IFG	MFG, IFG	7/7 (100)	VT, 6/7 (85.7)	4–61 (27.0)

*GMFT, gradient magnetic-field topography; iC, inferior central region; IFG, inferior frontal gyrus; ITG, inferior temporal gyrus; MFG, middle frontal gyrus; mT, medial temporal region; MTG, middle temporal gyrus; pITG, posterior part of inferior temporal gyrus; pSTG, posterior part of superior temporal gyrus; sC, superior central region; SFG, superior frontal gyrus; SMG, supramarginal gyrus; SPL, superior parietal lobule; STG, superior temporal gyrus; Tbase, basal area of temporal lobe; VT, voltage topography*.

**Figure 5:**
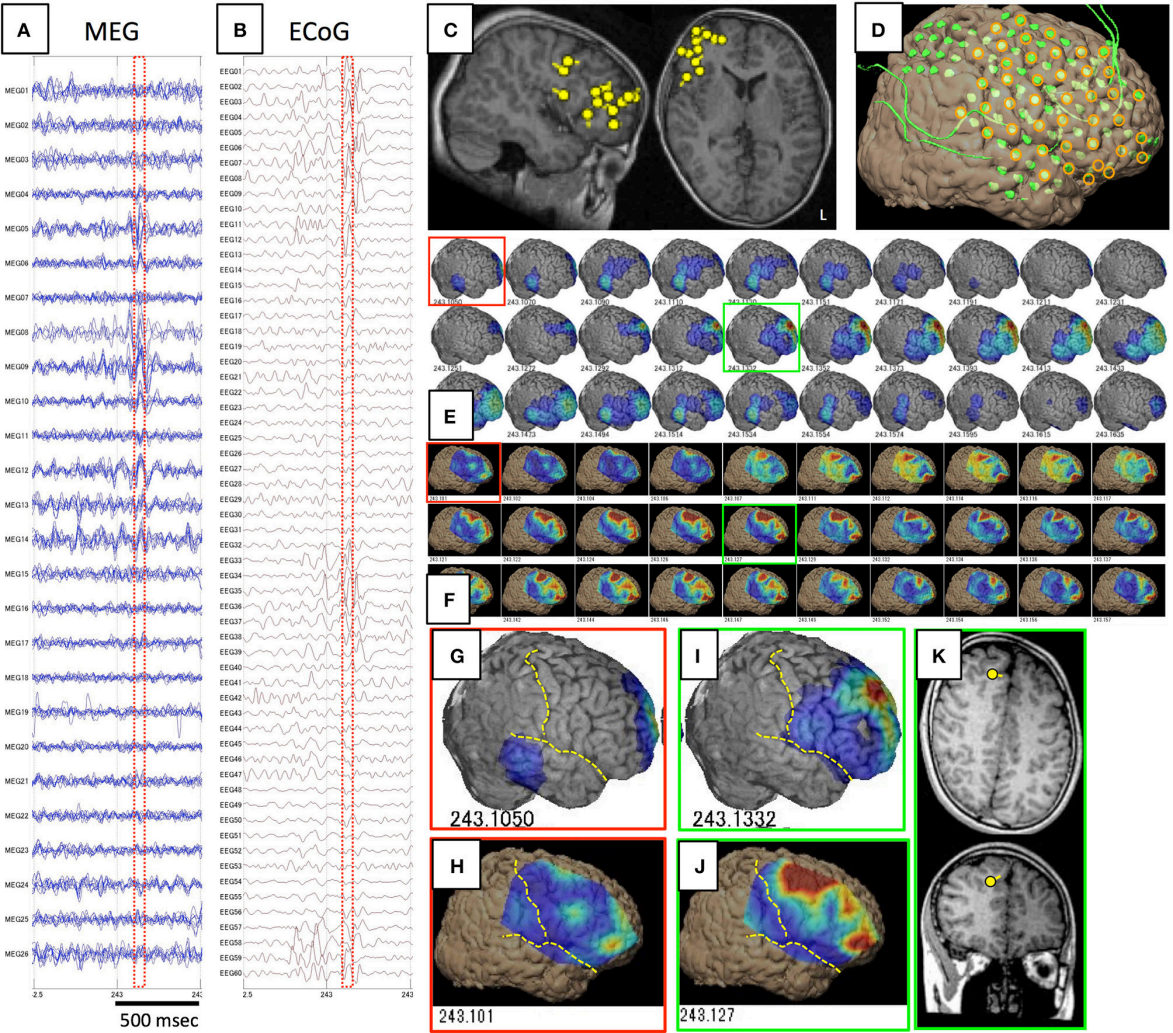
**Spike ME identified in Case 12**. **(A–D)** are the same as in Figure 1. Epileptic activity starts at the frontal tip (red squares) as demonstrated on both GMFT **(E,G)** and VT **(F,H)**. Spike activity propagates broadly to the superior, middle, and inferior frontal gyri (green squares), and activity is similarly reflected by both GMFT **(I)** and VT **(J)**. Yellow dotted lines indicate the central sulcus and Sylvian fissure. ECD is not estimated in the early phase (red squares), but instead in the late phase (green squares) in the superior frontal gyrus **(K)**, which corresponds to the center of activated areas of GMFT and VT.

In 65 of the 70 spike MEs (92.9%), activation areas on VT preceded those on GMFT. Time lags for total spike MEs ranged from 0 to 81 ms. Median time lag at onset between GMFT and VT in each patient ranged from 11.0 to 42.0 ms. Although some time lag was clearly evident, temporal changes in spatial propagation showed the same transitions (Figure 5).

## Discussion

We confirmed that the spatiotemporal changes in gradient magnetic-field of brain surface activities represented on GMFT were generally concordant with those of electrical potential distributions. We confirmed this hypothesis by simultaneously recording MEG and iEEG in patients with intractable epilepsy. These findings support the general utility of GMFT for evaluating epilepsy and possibly for investigation of various brain functions.

Because iEEG provides precise information on activated brain areas, simultaneous recordings of MEG and iEEG may validate the spatiotemporal accuracy of GMFT by direct comparison of both datasets. However, implantation of IEs is invasive. Patients with medically intractable epilepsy undergo resective surgery to treat epileptic seizures, and some of those cases require implantation of IEs to determine the area to be resected to improve epilepsy. Implantation of IEs offers an opportunity to clarify actual cortical activities. Moreover, epileptic spikes exhibit distinct spatiotemporal dynamics that are distinguishable from background activity, particularly in patients with localization-related epilepsy. Comparison with epileptic activities on iEEG is thus suitable to evaluate the spatiotemporal resolution of MEG. Few studies have undertaken simultaneous recording of MEG and iEEG to evaluate analytical methods (Mikuni et al., 1997; Oishi et al., 2002; Shigeto et al., 2002; Sutherling et al., 2002; Santiuste et al., 2008). All of those evaluated spike detection or accuracy of ECD, but not the spatial resolution of spatial filters used to determine the spatial distribution of cortical activity. To the best of our knowledge, this is the first report to examine the accuracy of spatial distribution analysis for MEG including various spatial filters.

### GMFT

GMFT is a unique method to examine the spatiotemporal dynamics of brain surface activities. GMFT is based on the characteristics of planar gradiometers. The maximum gradient magnetic-field picked up by planar gradiometers is located just above the electrical current source (Ahonen et al., 1993; Hämäläinen et al., 1993). The signal profile of a planar gradiometer differs markedly from that of a general magnetometer or axial gradiometer. The distribution of a general magnetic-field shows a dipole pattern, with influx and efflux of the magnetic field around the electrical current source, whereas the gradient magnetic-field of the planar gradiometer shows no such pattern. The principle of GMFT is simple, with sensors dropped vertically to the cortical surface, basal sensors with no underlying cortex projected to the nearest temporal base, and color-coded topographies drawn on the surface according to the magnitude of sensor signals. GMFT simply demonstrates the distribution of the gradient magnetic-field, so the results are visually comprehensible. Because of the simple method of generation in which the power of the gradient magnetic-field for each sensor is projected to the individual 3D-rendered MRI, GMFT does not need a solution to the ill-posed biomagnetic inverse problem. This means that there is no necessity for mathematically assumed constraints for the results of GMFT. Spatial filters including minimum norm estimation (Molins et al., 2008; Tanaka et al., 2010) or adaptive spatial filtering (Kirsch et al., 2006; Ishii et al., 2008) also evaluate spatial distributions. These methods can mathematically reconstruct source signals by mixing sensor signals with adequate weighting. This mixing process means that signals from noisy sensors influence the entire source space. Spatial filters therefore require adequate SNR from the intended data for physiologically reasonable source reconstruction (Bowyer et al., 2003). In contrast, GMFT is less influenced by SNR as compared to spatial filters, because of the lack of necessity for the mixing process mentioned above. Due to these characteristics of GMFT, we hypothesized that GMFT can faithfully reflect brain surface activity.

### Spatiotemporal accuracy of GMFT

This study demonstrated that GMFT depicted brain surface activity in a similar manner to that detected by VT, with concordance at the gyral unit level. Due to the nature of GMFT generation, detectability is limited to the lateral cortical surface. Another result of this study was that activity limited to the deep area of depth electrodes or at the temporal base was not reflected in MEG waves and was inevitably not detected by GMFT. This limitation means that the activated area on GMFT reflects only activity at the cortical surface, not in deep brain areas. This fact is crucial when interpreting the results of GMFT.

Slight time lag exists between GMFT and VT, and is considered to reflect the differences in the detection principles underlying MEG and iEEG. The detectability of MEG is attenuated in proportion to the square of the distance between the cortex and channel. Cortical activities away from the MEG sensor can also be masked by those nearer the sensor. Furthermore, MEG generally detects cortical activities of hidden sulcal banks, where currents arising from folded banks cancel fluxes in each other, while iEEG detects those of the superficial gyral surface without cancellation (Ochi et al., 2001). Although these factors might have affected the present results, the time lag was considered acceptable, and in accordance with a previous report describing propagation of interictal activity (Alarcon et al., 1994). That report described time delays up to 220 ms for intracerebral propagation. Our data showed a maximum time lag between GMFT and VT of 81 ms, and our GMFT evaluation method confirmed that MEG offers more precise temporal accuracy than previous reports.

### Application of GMFT

GMFT shows the ability for localization of epileptic activity. Spatiotemporal information from GMFT has a potential advantage of providing meaningful findings not demonstrated in conventional ECD analysis. Both spatial distributions and temporal changes are significant in evaluating epileptic activity. Epileptic spikes are usually provoked by some degree of simultaneous cortical activation. Even with interictal spikes, networking of brain areas is seen from onset to propagation (Alarcon et al., 1994; Merlet et al., 1996; Lantz et al., 2003). Investigations using IEs have demonstrated that the onset or earliest area of interictal activity considered as the generator of seizure are crucial, especially in surgical candidates (Alarcon et al., 1997; Hufnagel et al., 2000). Although onset of activity is important to localize the epileptic focus, ECD is disadvantageous for analyzing the onset of epileptic spikes corresponding to the rising phase of spikes. Because the rising phase of spikes shows low SNR, ECD cannot achieve localization with good statistical confidence, as represented by the goodness of fit. Conversely, GMFT can show spatial distributions even in phase with low SNR. Previous reports on the clinical usefulness of GMFT for patients with neocortical epilepsy have suggested the importance of the onset area of interictal spikes as represented by GMFT (Shirozu et al., 2010). The reliability of this hypothesis is supported by the spatiotemporal accuracy of GMFT. Cases may be seen in which the area of activity onset represented by GMFT deviates from the distribution of ECD. ECD shows only the center of activity at one time point, and usually analyzes the peak of spikes that may have already propagated to a broad area. Analysis of spike onset by GMFT provides an alternative source of information on epileptic networks, even with interictal spikes.

The ability to clarify the spatiotemporal dynamics of brain surface activity may also offer the potential to investigate various brain functions. Brain functions usually involve networks of various cortical areas. These networks are often investigated by IE or functional MRI (fMRI), as well-known and useful tools to explore brain functions that are widely used in basic neuroscience and clinical evaluations (Hirsch et al., 2000; Rutten et al., 2002; Detre, 2006). However, fMRI measures blood oxygenation level-dependent (BOLD) signals that reflect the blood flow associated with brain activity (Ogawa et al., 1990). These findings represent indirect results of actual brain activity, and some time lag exists between actual brain activity and the BOLD signal (Murayama et al., 2010). Examination using IEs is considered the best way to directly investigate actual brain activity. Numerous study using IEs to investigate brain functions have been conducted (Darcey et al., 2000; Ojemann et al., 2002; Matsumoto et al., 2004). However, this method is difficult to apply to everyone, due to the invasiveness of IE implantation. Using GMFT with its good spatiotemporal resolution, noninvasive MEG may facilitate the exploration of various brain functions.

### Limitations of this study

MEG shows advantages in the ability to measure whole-brain activity. Even though GMFT shows only activity of the lateral neocortex, GMFT is able to evaluate a large area of brain surface. However, iEEG can analyze limited brain areas where IEs have been implanted. The capacity of the EEG channels of a neuromagnetometer (Neuromag, in this study) is also limited to 60 channels. Because the entire brain surface was not covered by IEs, VT could not confirm the total dynamics of GMFT. Another limitation influencing this study was the sensitivity of MEG. In this study, localized spikes detected only on iEEG were more common than those detected only on MEG or simultaneously on both. This finding implies that iEEG is more sensitive to localized cortical activity. Moreover, magnetic fields might be distorted by various factors, such as IE implantation. These factors include electrode thickness, existence of epi- or subdural hematoma, and pressure effects of IE itself. IE may exert pressure on the underlying cortices and thus affect their excitability, conductivity, and synchronization. The VT of iEEG in this study thus may have been insufficient to prove the accuracy of whole GMFT in the strictest sense. However, we thought that the tendencies of spatiotemporal dynamics offered an acceptable reflection of the accuracy of GMFT. The accuracy of ECD has been reported at the level of millimeters, whereas that of GMFT appears to be at the gyral level given the results of this study. Despite such differences in accuracy, we consider the accuracy to the gyral unit level as sufficient for evaluating brain function or epileptic networks. Most brain functions are organized with some extent of brain constructed by gyral units, and resective surgery for focal epilepsy is often performed by gyral resection (gyrectomy). From this perspective, accuracy to the gyral unit level is acceptable for these evaluations.

## Conclusions

This study demonstrated that GMFT offers good spatiotemporal accuracy, as confirmed by simultaneous recordings of MEG and iEEG. Spatial accuracy was at the gyral unit level and temporal accuracy was considered acceptable. Noninvasive MEG may offer a valuable contribution to clinical evaluations using GMFT.

## Author contributions

HS conducted the experiments, analyzed the data and wrote the first version of manuscript. AH supported and supervised the methodology, the results of this experiments and the manuscript. HM supported the experiments, acquisition of data, and surgery. SK and MF supervised this study and manuscript. YI, YN, and TH supported the experiments and acquisition of data.

## Funding

This study was supported in part by a Health Labour Sciences Research Grant from the Ministry of Health, Labour, and Welfare of Japan.

### Conflict of interest statement

The authors declare that the research was conducted in the absence of any commercial or financial relationships that could be construed as a potential conflict of interest.

## References

[B1] Ahonen A. I. Hämäläinen M. S. Ilmoniemi R. J. Kajola M. J. Knuutila J. E. T. Simola J. T.. (1993). Sampling theory for neuromagnetic detector arrays. IEEE Trans. Biomed. Eng. 40, 859–869. 10.1109/10.245606 8288276

[B2] Alarcon G. Guy C. N. Binnie C. D. Walker S. R. Elwes R. D. Polkey C. E. (1994). Intracerebral propagation of interictal activity in partial epilepsy: implications for source localisation. J. Neurol. Neurosurg. Psychiatry 57, 435–449. 8163992 10.1136/jnnp.57.4.435 PMC1072872

[B3] Alarcon G. Seoane J. J. G. Binnie C. D. Miguel M. C. M. Juler J. Polkey C. E.. (1997). Origin and propagation of interictal discharges in the acute electrocorticogram. Implications for pathophysiology and surgical treatment of temporal lobe epilepsy. Brain 120(Pt 12), 2259–2282. 9448581 10.1093/brain/120.12.2259

[B4] Baumgartner C. Doppelbauer A. Deecke L. Barth D. S. Zeitlhofer J. Lindinger G.. (1991). Neuromagnetic investigation of somatotopy of human hand somatosensory cortex. Exp. Brain Res. 87, 641–648. 10.1136/jnnp.57.4.435 1783032

[B5] Bowyer S. M. Mason K. Tepley N. Smith B. Barkley G. L. (2003). Magnetoencephalographic validation parameters for clinical evaluation of interictal epileptic activity. J. Clin. Neurophysiol. 20, 87–93. 10.1097/00004691-200304000-00001 12766680

[B6] Darcey T. M. Hughes H. C. Barkan H. I. Saykin A. J. (2000). Preoperative functional mapping using intracranial EEG activation methods. Adv. Neurol. 84, 331–341. 11091877

[B7] Detre J. A. (2006). Clinical applicability of functional MRI. J. Magn. Reson. Imaging 23, 808–815. 10.1097/00004691-200304000-00001 16649200

[B8] Gharib S. Sutherling W. W. Nakasato N. Barth D. S. Baumgartner C. Alexopoulos N.. (1995). MEG and ECoG localization accuracy test. Electroencephalogr. Clin. Neurophysiol. 94, 109–114. 7532571 10.1016/0013-4694(94)00276-q

[B9] Gow D. W. Jr. Caplan D. N. (2012). New levels of language processing complexity and organization revealed by granger causation. Front. Psychol. 3:506. 10.3389/fpsyg.2012.00506 23293611 PMC3536267

[B10] Hämäläinen M. Hari R. Ilmoniemi R. J. Knuutila J. Lounasmaa O. V. (1993). Magnetoencephalography-theory, instrumentation, and application to noninvasive studies of the working human brain. Rev. Mod. Phys. 65, 413–497.

[B11] Hashizume A. Iida K. Shirozu H. Hanaya R. Kiura Y. Kurisu K.. (2007). Gradient magnetic-field topography for dynamic changes of epileptic discharges. Brain Res. 1144, 175–179. 10.1103/RevModPhys.65.413 17331481

[B12] Hirsch J. Ruge M. I. Kim K. H. S. Correa D. D. Victor J. D. Relkin N. R.. (2000). An integrated functional magnetic resonance imaging procedure for preoperative mapping of cortical areas associated with tactile, motor, language, and visual functions. Neurosurgery 47, 711–721. discussion: 721–722. 10.1227/00006123-200009000-00037 10981759

[B13] Hufnagel A. Dümpelmann M. Zentner J. Schijns O. Elger C. E. (2000). Clinical relevance of quantified intracranial interictal spike activity in presurgical evaluation of epilepsy. Epilepsia 41, 467–478. 10.1111/j.1528-1157.2000.tb00191.x 10756415

[B14] Ishii R. Canuet L. Ochi A. Xiang J. Imai K. Chan D.. (2008). Spatially filtered magnetoencephalography compared with electrocorticography to identify intrinsically epileptogenic focal cortical dysplasia. Epilepsy Res. 81, 228–232. 10.1016/j.eplepsyres.2008.06.006 18672350

[B15] Kirsch H. E. Robinson S. E. Mantle M. Nagarajan S. (2006). Automated localization of magnetoencephalographic interictal spikes by adaptive spatial filtering. Clin. Neurophysiol. 117, 2264–2271. 10.1016/j.clinph.2006.06.708 16893680

[B16] Knowlton R. C. Laxer K. D. Aminoff M. J. Roberts T. P. Wong S. T. Rowley H. A. (1997). Magnetoencephalography in partial epilepsy: clinical yield and localization accuracy. Ann. Neurol. 42, 622–631. 9382474 10.1002/ana.410420413

[B17] Lantz G. Spinelli L. Seeck M. de Peralta Menendez R. G. Sottas C. C. Michel C. M. (2003). Propagation of interictal epileptiform activity can lead to erroneous source localizations: a 128-channel EEG mapping study. J. Clin. Neurophysiol. 20, 311–319. 10.1097/00004691-200309000-00003 14701992

[B18] Matsumoto R. Nair D. R. LaPresto E. Najm I. Bingaman W. Shibasaki H.. (2004). Functional connectivity in the human language system: a cortico-cortical evoked potential study. Brain 127, 2316–2330. 10.1093/brain/awh246 15269116

[B19] Merlet I. García-Larrea L. Grégoire M. C. Lavenne F. Mauguière F. (1996). Source propagation of interictal spikes in temporal lobe epilepsy. Correlations between spike dipole modelling and [18F]fluorodeoxyglucose PET data. Brain 119(Pt 2), 377–392. 8800934 10.1093/brain/119.2.377

[B20] Mikuni N. Nagamine T. Ikeda A. Terada K. Taki W. Kimura J.. (1997). Simultaneous recording of epileptiform discharges by MEG and subdural electrodes in temporal lobe epilepsy. Neuroimage 5, 298–306. 9345559 10.1006/nimg.1997.0272

[B21] Molins A. Stufflebeam S. M. Brown E. N. Hämäläinen M. S. (2008). Quantification of the benefit from integrating MEG and EEG data in minimum ℓ*2*-norm estimation. Neuroimage 42, 1069–1077. 10.1016/j.neuroimage.2008.05.064 18602485

[B22] Murayama Y. Bießmann F. Meinecke F. C. Müller K.-R. Augath M. Oeltermann A.. (2010). Relationship between neural and hemodynamic signals during spontaneous activity studied with temporal kernel CCA. Magn. Reson. Imaging 28, 1095–1103. 10.1016/j.mri.2009.12.016 20096530

[B23] Nakasato N. Fujita S. Seki K. Kawamura T. Matani A. Tamura I.. (1995). Functional localization of bilateral auditory cortices using an MRI-linked whole head magnetoencephalography (MEG) system. Electroencephalogr. Clin. Neurophysiol. 94, 183–190. 7536153 10.1016/0013-4694(94)00280-x

[B24] Nakasato N. Yoshimoto T. (2000). Somatosensory, auditory, and visual evoked magnetic fields in patients with brain diseases. J. Clin. Neurophysiol. 17, 201–211. 10.1097/00004691-200003000-00009 10831111

[B25] Ochi A. Otsubo H. Sharma R. Hunjan A. Rutka J. T. Chuang S. H.. (2001). Comparison of electroencephalographic dipoles of interictal spikes from prolonged scalp video-electroencephalography and magnetoencephalographic dipoles from short-term recording in children with extratemporal lobe epilepsy. J. Child Neurol. 16, 661–667. 10.1177/088307380101600907 11575607

[B26] Ogawa S. Lee T. M. Kay A. R. Tank D. W. (1990). Brain magnetic resonance imaging with contrast dependent on blood oxygenation. Proc. Natl. Acad. Sci. U.S.A. 87, 9868–9872. 2124706 10.1073/pnas.87.24.9868 PMC55275

[B27] Oishi M. Kameyama S. Masuda H. Tohyama J. Kanazawa O. Sasagawa M.. (2006). Single and multiple clusters of magnetoencephalographic dipoles in neocortical epilepsy: significance in characterizing the epileptogenic zone. Epilepsia 47, 355–364. 10.1111/j.1528-1167.2006.00428.x 16499760

[B28] Oishi M. Otsubo H. Kameyama S. Morota N. Masuda H. Kitayama M.. (2002). Epileptic spikes: magnetoencephalography versus simultaneous electrocorticography. Epilepsia 43, 1390–1395. 10.1046/j.1528-1157.2002.10702.x 12423390

[B29] Ojemann J. G. Ojemann G. A. Lettich E. (2002). Cortical stimulation mapping of language cortex by using a verb generation task: effects of learning and comparison to mapping based on object naming. J. Neurosurg. 97, 33–38. 10.3171/jns.2002.97.1.0033 12134930

[B30] Onishi H. Sugawara K. Yamashiro K. Sato D. Suzuki M. Kirimoto H.. (2013). Neuromagnetic activation following active and passive finger movements. Brain Behav. 3, 178–192. 10.1002/brb3.126 23531918 PMC3607158

[B31] Otsubo H. Ochi A. Elliott I. Chuang S. H. Rutka J. T. Jay V.. (2001a). MEG predicts epileptic zone in lesional extrahippocampal epilepsy: 12 pediatric surgery cases. Epilepsia 42, 1523–1530. 10.1046/j.1528-1157.2001.16701.x 11879362

[B32] Otsubo H. Shirasawa A. Chitoku S. Rutka J. T. Wilson S. B. Snead O. C. (2001b). Computerized brain-surface voltage topographic mapping for localization of intracranial spikes from electrocorticography. Technical note. J. Neurosurg. 94, 1005–1009. 10.3171/jns.2001.94.6.1005 11409502

[B33] Pataraia E. Baumgartner C. Lindinger G. Deecke L. (2002). Magnetoencephalography in presurgical epilepsy evaluation. Neurosurg. Rev. 25, 141–159. discussion: 160–161. 10.1007/s10143-001-0197-2 12135228

[B34] Rutten G. J. M. Ramsey N. F. van Rijen P. C. Noordmans H. J. van Veelen C. W. M. (2002). Development of a functional magnetic resonance imaging protocol for intraoperative localization of critical temporoparietal language areas. Ann. Neurol. 51, 350–360. 10.1002/ana.10117 11891830

[B35] Santiuste M. Nowak R. Russi A. Tarancon T. Oliver B. Ayats E.. (2008). Simultaneous magnetoencephalography and intracranial EEG registration: technical and clinical aspects. J. Clin. Neurophysiol. 25, 331–339. 10.1097/WNP.0b013e31818e7913 18997623

[B36] Seki K. Nakasato N. Fujita S. Hatanaka K. Kawamura T. Kanno A.. (1996). Neuromagnetic evidence that the P100 component of the pattern reversal visual evoked response originates in the bottom of the calcarine fissure. Electroencephalogr. Clin. Neurophysiol. 100, 436–442. 8893661

[B37] Shigeto H. Morioka T. Hisada K. Nishio S. Ishibashi H. Kira D.. (2002). Feasibility and limitations of magnetoencephalographic detection of epileptic discharges: simultaneous recording of magnetic fields and electrocorticography. Neurol. Res. 24, 531–536. 10.1179/016164102101200492 12238617

[B38] Shirozu H. Iida K. Hashizume A. Hanaya R. Kiura Y. Kurisu K.. (2010). Gradient magnetic-field topography reflecting cortical activities of neocortical epilepsy spikes. Epilepsy Res. 90, 121–131. 10.1016/j.eplepsyres.2010.04.002 20451350

[B39] Spencer S. S. (2002). Neural networks in human epilepsy: evidence of and implications for treatment. Epilepsia 43, 219–227. 10.1046/j.1528-1157.2002.26901.x 11906505

[B40] Stefan H. Hummel C. Scheler G. Genow A. Druschky K. Tilz C.. (2003). Magnetic brain source imaging of focal epileptic activity: a synopsis of 455 cases. Brain 126, 2396–2405. 10.1093/brain/awg239 12876149

[B41] Sutherling W. W. Akhtari M. Mamelak A. N. Mosher J. Arthur D. Sands S.. (2002). Dipole localization of human induced focal afterdischarge seizure in simultaneous magnetoencephalography and electrocorticography. Brain Topogr. 14, 101–116. 10.1023/A:1012940812742 11797809

[B42] Tanaka N. Hämäläinen M. S. Ahlfors S. P. Liu H. Madsen J. R. Bourgeois B. F.. (2010). Propagation of epileptic spikes reconstructed from spatiotemporal magnetoencephalographic and electroencephalographic source analysis. Neuroimage 50, 217–222. 10.1046/j.1528-1157.2001.16701.x 20006721 PMC2862548

